# Evaluation of attitudes of university students towards artificial intelligence using data mining methods

**DOI:** 10.1038/s41598-025-25748-0

**Published:** 2025-11-25

**Authors:** Suleyman Alpaslan Sulak

**Affiliations:** Ahmet Kelesoglu Educational Faculty, Necmettin Erbakan University, Konya, Türkiye

**Keywords:** Artificial intelligence, Data mining, Attitude, SVM, CatBoost, Random forrest, Engineering, Mathematics and computing

## Abstract

This study analyzes university students’ attitudes towards artificial intelligence. Within the scope of the research, the data obtained from 1379 students through scale application were classified into three classes as “Insufficient”, “Sufficient” and “Strongly Sufficient” according to their attitudes towards artificial intelligence. The data was classified by data mining methods. For this purpose, MLP, Decision Tree, KNN, XGBoost, Random Forest, CatBoost and SVM algorithms were used. The performance of the model was evaluated with a 5-fold cross-validation method. For each algorithm, basic metrics such as accuracy, precision, recall and F1 score were calculated and the classification performance was compared. According to the results, the highest F1-Score accuracy rate was 95.52% for the SVM algorithm. This was followed by CatBoost (93.66%), Random Forest (92.56%) and XGBoost (92.36%). The lowest success rates were observed in MLP (81.87%) and Decision Tree (82.72%) models. Confusion matrices revealed a tendency for frequent confusion with other classes, especially in the Strongly Sufficient class. The study concluded that advanced classification algorithms provide powerful and reliable tools for analyzing students’ attitudes towards artificial intelligence. These findings may contribute to the development of educational policies and strategies for AI literacy.

## Introduction

As artificial intelligence (AI) technologies have rapidly evolved, many sectors have undergone radical changes it is also reshaping teaching processes and student experiences in higher education. This transformation has made it even more critical to understand individuals’ relationship to AI technologies and to study students’ attitudes in this context^1^. University students stand out as a generation that will interact directly with AI technologies. This is not only in the current educational environment, but also in their future professional lives. Therefore, uncovering students’ perceptions, attitudes, expectations and concerns towards AI plays a significant role in guiding educational policies, teaching strategies and technological integration^2,3^.

As a result of the integration of AI in education, students have the opportunity to learn at their own pace and the teaching process can be restructured^4^. Students know the risks and benefits of these technologies. Literature often shows that students accept that AI will play an important role in their field in the future^3,5^. Furthermore, correlational studies show that there may be significant relationships between AI use and academic performance^6^. In quantitative research, Likert-type scales are common, and these tools stand out as a valuable tool for understanding students’ perceptions, frequency of use, and evaluation of AI technologies^7^.

AI integration into higher education curricula offers many advantages such as enriching course materials, individualizing the learning process and increasing student achievement. However, ethical frameworks need to be established for the responsible use of this technology^8^. Issues such as academic integrity, algorithmic biases, data privacy, and over-reliance on AI are among the potential threats that may conflict with education’s core values^9,10^.

Integrations that do not distinguish between students’ personal and pedagogical use of AI may not adequately respond to individual learning preferences. Institutional policies and ethical governance mechanisms play an important role here^11^. It is important for educators to emphasize the primacy of human expertise and argue that artificial intelligence should be viewed as a supportive tool^12^.

University students vary in their emotional reactions towards AI. While some see this technology as a means of efficiency, others associate it with concerns such as unemployment, injustice or invasion of privacy^13^. Therefore, there should be open discussion about AI in educational settings and space for student views. In particular, there should be transparency about how student data is collected, stored and analysed^14^.

AI technologies must be used effectively, ethically, and pedagogically in order to transform university students’ learning experiences. Analysing students’ attitudes and perceptions towards AI can provide important contributions to shaping teaching strategies, curriculum designs and educational policies. In this context, studies in which quantitative and qualitative research methods are used together will enable the development of more holistic and sustainable educational strategies.

Educators, policy makers and institutions should determine ethical principles for AI technologies use in educational settings. They should guide these technologies in line with transparency and fairness principles. With this approach, balanced and responsible AI integration will be possible. This will contribute to both the individual and professional development of students as professionals of the future. University students’ attitudes towards AI will directly affect today’s education system and tomorrow’s social structure.

This study contributes to the literature in several novel ways. First, it utilizes a large-scale dataset of 1,379 university students from Turkey, offering a unique, context-specific perspective on attitudes towards artificial intelligence (AI). Second, a multi-algorithm approach—including MLP, Decision Tree, KNN, XGBoost, Random Forest, CatBoost, and SVM—allows for a comprehensive comparison of classification performance. Third, class-level analyses via confusion matrices provide detailed insights into misclassification patterns, particularly in the ‘Strongly Sufficient’ category, highlighting the complexity of students’ attitudes. Finally, the study offers practical implications for AI literacy programs and educational policy development, bridging methodological rigor with actionable educational insights.

### Literature review and related works

Studies evaluating student attitudes towards AI offer various methodological approaches in different contexts. Jang, Choi, and Kim; developed a valid and reliable scale consisting of five dimensions: “Fairness”, “Transparency”, “Do No Harm”, “Responsibility”, and “Privacy” to measure student attitudes towards AI ethics^15^. The study was conducted with 1,076 Korean university students and the psychometric properties of the scale were tested with explanatory and confirmatory factor analyses. On the other hand, Pinto dos Santos et al. examined the attitudes of 263 medical students from three different universities in Germany towards artificial intelligence, especially its use in radiology^16^. Students believe that AI can complement medicine, but they do not favor its replacement by humans. A significant number of respondents stated that AI should be incorporated into medical education. Ghotbi, Ho and Mantello analysed the views of 228 students studying at an international university in Japan on the ethical issues of artificial intelligence, with data collected in writing on nine ethical topics identified by the World Economic Forum^17^. In this study, which was conducted with text mining and sentiment analysis methods, it was determined that 65% of students saw unemployment as the most important ethical problem related to artificial intelligence, followed by emotional artificial intelligence and inequality. When the three studies are evaluated together, students generally develop positive attitudes towards artificial intelligence; however, ethical concerns, uncertainties about their professional future and interdisciplinary knowledge differences shape these attitudes. Some similar studies in the literature are summarized in Table 1. This study will examine the evaluation of university students’ attitudes towards artificial intelligence using data mining methods.

**Table 1:** Related Works.

	Title	Sample	Technique	Finding	Reference
1	An Investigation of University Students’ Attitudes towards Artificial Intelligence Ethics	University students	Mixed Quantitative-Qualitative	Female and Education Faculty students were found to be more sensitive to digital ethics, standing out in areas such as transparency, privacy, and legal compliance.	^18^
2	Adaptation and validation of an instrument to measure university students’ attitudes towards artificial intelligence	University students	Quantitative	The survey was highly reliable in both its original and proposed structures.	^19^
3	Factors Affecting the Adoption of AI-Based Applications in Higher Education: An Analysis of Teachers’ Perspectives Using Structural Equation Modeling	Teachers	Structural Equation Model	The study developed a model explaining 70.4% of teachers’ intentions to use AI-based applications, highlighting ATU, PEU, and subjective norms as key predictors.	^20^
4	ChatGPT usage and attitudes are driven by perceptions of usefulness, ease of use, risks, and psycho-social impact: a study among university students in the UAE	University students	Online survey	ChatGPT is widely used among university students in the UAE, with perceived usefulness, low risk, and positive attitudes playing a significant role in its adoption.	^21^
5	Pre-service teachers’ attitudes towards artificial intelligence and its integration into EFL teaching and learning	University students	Online survey	While Slovak pre-service English teachers believe AI can contribute to education, they also express concerns about their lack of knowledge and the potential impact on teaching roles.	^22^
6	A multinational study on the factors influencing university students’ attitudes and usage of ChatGPT	University students	Online survey	Positive attitudes toward ChatGPT are shaped by individual, technological, and environmental factors, and the “TAME-ChatGPT” scale has been validated for measuring these attitudes.	^23^
7	University Students’ Attitudes and Perceptions towards AI Tools: Implications for Sustainable Educational Practices	University students	ANOVA	Students acknowledge AI’s efficiency but are cautious about its impact on learning quality and academic integrity, emphasizing the need for balanced and responsible integration.	^24^

## Materials and methods

**Fig. 1:**
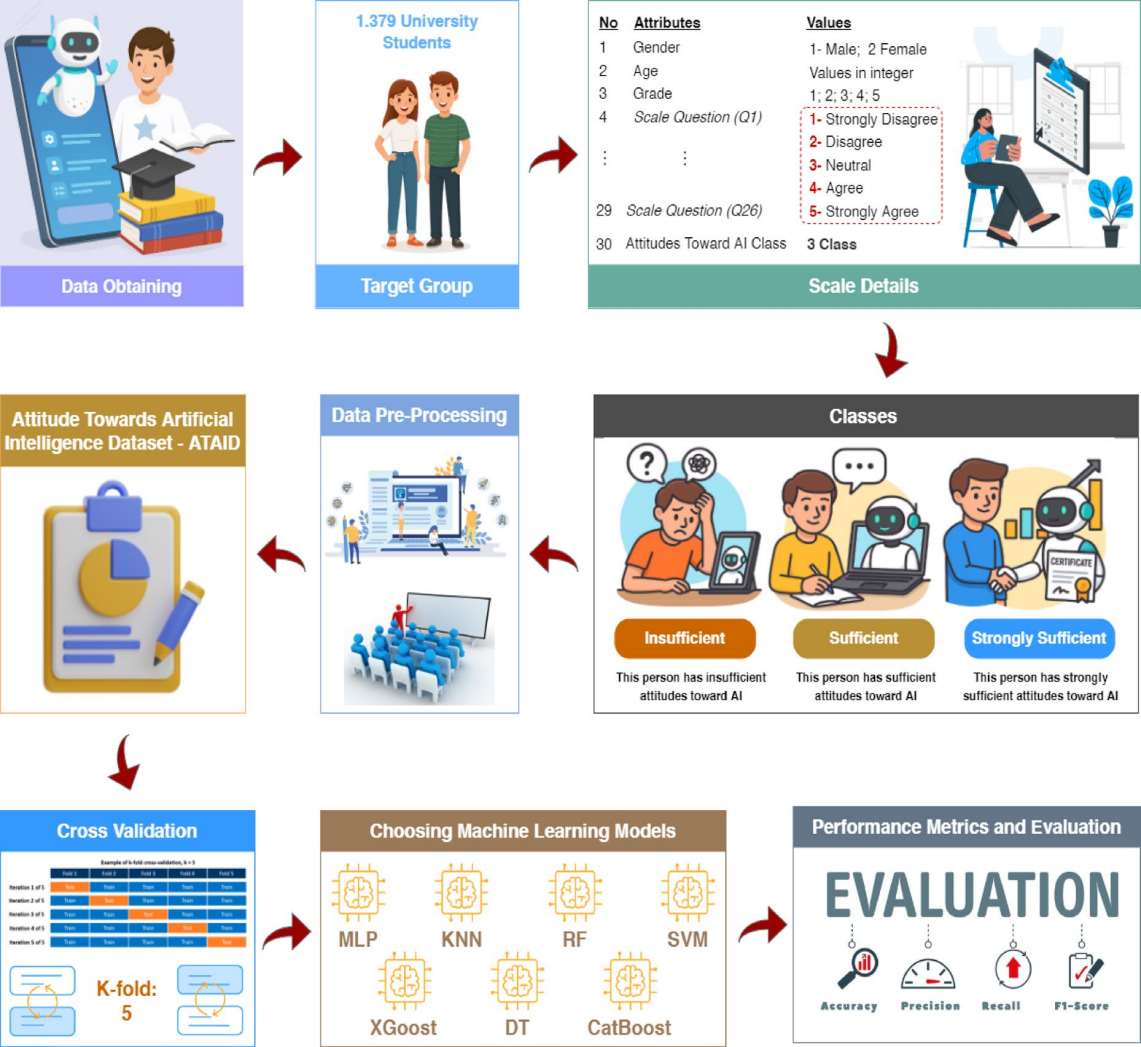
Flow diagram of this study.

In this article, it is aimed at evaluating university students’ attitudes towards artificial intelligence with data mining methods. Within the research, the SATAI scale developed by Suh and Ahn was used^25^. Data were obtained from 1379 adults and a dataset was created. In this section, dataset, confision matrix and performance metrics, cross validation, data mining methods (MLP, DT, KNN XGBoost, RF, CatBoost and SVM) and Data mining method parameters are mentioned. Flow diagram of this study is shown in Fig. 1.

The study was approved by the Ethics Committee for Social and Behavioral Sciences at Necmettin Erbakan University (Protocol No: 2025 − 280) and conducted in accordance with the relevant guidelines and regulations, as well as the principles of the Declaration of Helsinki.

### Attitude towards artificial intelligence dataset

A total of 1379 individuals living in Turkey completed an online survey administered by the authors. In the created dataset, 29 variables are capable of determining attidutes toward artificial intelligence. Informed consent was obtained from all participants prior to their involvement in the study.

At the beginning of the data collection process, demographic questions were asked, followed by 29 questions about Attitude Towards Artificial Intelligence, culminating in an additional feature indicating whether the individual had an Attitude Towards Artificial Intelligence. This resulted in a dataset comprising 29 variables. We collected data from 1379 university students across Turkey between April 14, 2025, and April 27, 2025.

Data distributions of the features of the Attitude Towards Artificial Intelligence Dataset, including demographic data and answers to the Attitude Towards Artificial Intelligence questions, are shown in Table 2.

**Table 2:** ATAID data set.

No	Attributes	Values
1	Gender	1-Male; 2-Feale
2	Age	Values in integer
3	Grade	1; 2; 3; 4; 5
4	Q1 I think that it is important to learn about AI in school.	1-Strongly disagree 2-Disagree 3-Neutral 4-Agree 5-Strongly agree
5	Q2 AI class is important.	
6	Q3 I think that lessons about AI should be taught in school.	
7	Q4 I think every student should learn about AI in school	
8	Q5 AI is very important for developing society.	
9	Q6 I think AI makes people’s lives more convenient.	
10	Q7 AI is related to my life	
11	Q8 I will use AI to solve problems in daily life.	
12	Q9 AI helps me solve problems in real life.	
13	Q10 I will need AI in my life in the future.	
14	Q11 AI is necessary for everyone.	
15	Q12 AI produces more good than bad.	
16	Q13 AI is worth studying.	
17	Q14 I think that most jobs in the future will require knowledge related to AI.	
18	Q15 I want to work in the field of AI.	
19	Q16 I will choose a job in the field of AI.	
20	Q17 I would participate in a club related to AI if there was one.	
21	Q18 I like using objects related to AI.	
22	Q19 It is fun to learn about AI.	
23	Q20 I want to continue learning about AI.	
24	Q21 I’m interested in AI-related TV programs or online videos.	
25	Q22 I want to make something that makes human life more convenient through AI.	
26	Q23 I am interested in the development of AI.	
27	Q24 It is interesting to use AI.	
28	Q25 I think that there should be more class time devoted to AI in school.	
29	Q26 I think I can handle AI well.	
**30**	**Attitudes Toward AI Class**	**1- Insufficient (This person has insufficient attitudes toward AI)**
		**2- Sufficient (This person has sufficient attitudes toward AI)**
		**3- Strongly sufficient (This person has strongly sufficient attitudes toward AI)**

In this study, we utilized a structured dataset consisting of 33 numerical features with a moderate sample size (*n* = 1,379). Among the participants, 210 were classified as ‘Insufficient’, 502 as ‘Sufficient’, and 667 as ‘Strongly Sufficient’. Although deep learning algorithms have demonstrated great success in complex and high-dimensional tasks, traditional machine learning methods were preferred due to their computational efficiency and interpretability. Deep neural networks may risk overfitting on limited data and require higher computational resources without necessarily improving performance. Therefore, classical algorithms such as decision trees and support vector machines were selected for their balance of accuracy, transparency, and suitability for tabular data.

### Confusion matrix and performance metrics

In order to evaluate the performance of classification algorithms, confusion matrixes are frequently used. It provides a tabular representation of the relationship between the model predicted classes and the actual classes and presents the results in detail, divided into four main categories: true positive (TP), true negative (TN), false positive (FP) and false negative (FN)^26,27^. As a rule, the rows of the matrix represent the actual classes, and the columns represent the predicted classes^28^. While an ideal model contains only values on the diagonal, in practical applications, off-diagonal values reflect the model’s errors and provide important insights^29^.

This structure allows us to calculate not only the overall accuracy rate but also more detailed performance measures such as accuracy, precision, recall and F1 score^30^. While true positives and true negatives reflect successful predictions of the model, false positives (Type I error) refer to cases where the model predicts a positive state when it is actually negative, and false negatives (Type II error) refer to cases where the model misses a positive state^31,32^. Detailed analysis of the confusion matrix is critical, especially when data sets are unbalanced, as relying on accuracy alone can lead to misleading results^33,34^. Furthermore, normalizing the matrix by the number of samples allows fair comparisons between different data sets^35^. In scenarios where the costs of different types of errors are not equal, the findings from the confusion matrix provide important contributions to decision makers in processes such as model selection, threshold adjustments and feature engineering^36^.

Model evaluation is a fundamental process used in machine learning to analyse a model’s performance^37^. Accuracy, precision, recall, and F1 scores are some of the most commonly used evaluation metrics in classification problems^30,38^.

**Accuracy**, is the ratio of correctly classified samples to the total number of samples and reflects the overall success^33^.

**Precision**, indicates how many of the model’s positive predictions are correct, i.e. the ratio of true positives to total positive predictions^39^.

**Recall or Sensitivity**, it expresses how many of the actually positive samples were predicted correctly; it is the ratio of true positives to the sum of true and false negatives^40^.

**F1 score**, By taking the harmonic mean of precision and sensitivity, it provides an evaluation that balances these two metrics. It stands out as a healthy performance measure, especially in unbalanced data sets where focusing on one metric is insufficient^41^.

### Cross validation

Cross-validation stands out as a fundamental technique in statistical model evaluation and provides a robust method for assessing how well statistical analysis results can be generalized to an independent dataset^42^. When prediction is the main goal, it is particularly useful as it helps predict in real-world scenarios how accurate a predictive model will be^43^. Cross-validation is used to test the algorithm with data it has not yet encountered, thus achieving a more realistic evaluation procedure and accuracy rate^44^. This technique is particularly valuable in cases where the available dataset is so inadequate that it cannot be divided into training and testing sets without losing statistical power. In short, cross-validation is a method of assessing a model’s ability to predict new data that is not used to predict it and helps to measure the generalizability and robustness of the model^45^.

**Fig. 2:**
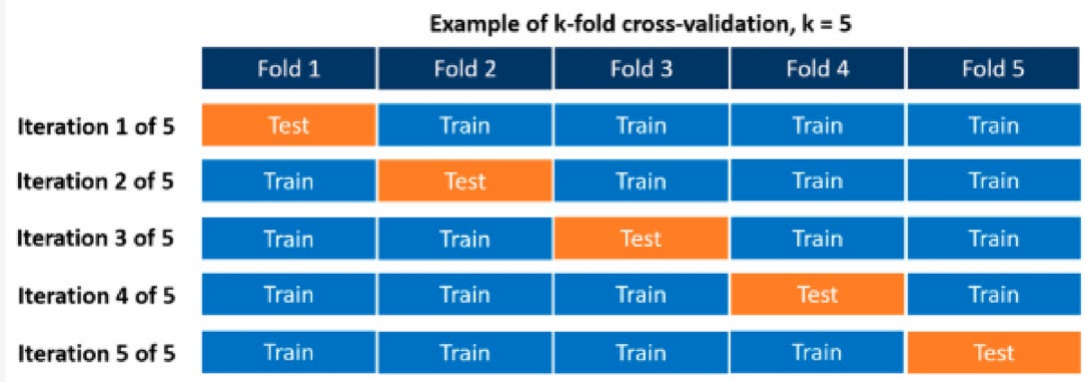
5-fold cross validation.

Cross-validation involves splitting available data into multiple subsets and using some subsets to train the model and the rest to evaluate its performance^46^. There are several repetitions of this process. During each iteration, a different layer of data is reserved for validation, and the remaining k-1 layers are used to train the model^47^. In this way, the model is trained and validated on all data points, which allows us to obtain an out-of-sample error estimate. Figure 2 shows the 5-fold cross validation used in this study^48^.

### Data mining methods

In this study, various data mining methods were used to effectively analyse and classify the dataset. Each method offers its own advantages in prediction accuracy, interpretability and computational efficiency. By applying different algorithms, it is aimed to compare their performance and determining the most appropriate model for the problem of interest. The selected methods represent both traditional and modern approaches in machine learning, ranging from tree-based models to artificial neural networks and support vector machines. Brief descriptions of the seven data mining techniques used in this research are given below.

#### Multilayer perceptron (MLP)

MLPs are powerful models that form the basis of artificial neural networks and have been used successfully for data mining due to their ability to learn complex patterns and relationships in high-dimensional datasets^49,50^. This structure, which consists of an input layer, one or more hidden layers and an output layer, processes information with weighted connections between layers. Since MLP has the ability to approximate any continuous function, it is suitable for various data mining applications such as classification, regression and clustering. The model’s performance is affected by configuration parameters such as transfer function, learning rate and number of layers, and the training process is usually performed by backpropagation^51,52^. Moreover, for the training process to be efficient, it is crucial to normalize the input data with an appropriate scaling method^53^.

#### Decision tree (DT)

Decision trees are an intuitive and effective approach for both classification and regression tasks in supervised learning. It works by iteratively subdividing the data according to certain decision rules^54^. Thanks to their nonparametric structure, they can be applied without making strict assumptions about data distribution^55^. Decision trees have a hierarchical structure where each internal node contains a feature-based decision rule and the leaf nodes contain the result^56^. The algorithm aims to reach the target value of an object based on its observations^57^. When creating a tree structure, the most informative feature is selected as the root node. Criteria such as information gain, Gini purity or variance reduction are used in this selection^58^. This feature is then used to divide the data and the tree is formed by repeating the same process for each subgroup^59^.

#### K-Nearest neighbor (KNN)

The K-Nearest Neighbor (KNN) algorithm is an important supervised learning technique widely used in classification and regression tasks. KNN determines the class or value of a new data point by looking at the labels or values of its ‘k’ nearest neighbors in the dataset^60^. KNN is an instance-based and non-parametric algorithm, meaning that it does not build models during the training phase; instead, it bases predictions on the entire dataset, which makes it sensitive to the structure of the data^61^. The algorithm calculates the similarity between data points, usually using distance metrics such as Euclidean distance, to determine the influence of neighbouring points^62^. Its simple structure and easy applicability make KNN a popular choice for small and medium-sized datasets^63^. K-Nearest Neighbour is a classical nonparametric classification algorithm and is preferred in pattern recognition due to its simplicity, efficiency and intuitive nature^64^.

#### Extreme gradient boosting (XGBoost)

Extreme Gradient Boosting (XGBoost) is a powerful machine learning algorithm that provides high performance in classification and regression tasks. It is widely preferred due to its ability to efficiently handle complex datasets and deliver superior results. XGBoost is an advanced version of the gradient boosting framework and uses an ensemble of decision trees to improve weak learners respectively, resulting in higher accuracy. Each new tree is designed to correct the errors of the previous trees, improving the model’s performance. The principle of gradient descent captures complex patterns in data by optimizing the model parameters^65,66^. This process is supported by L1 and L2 regularization techniques to prevent overfitting and increase the generalization ability of the model^67^. One of XGBoost’s strengths is that it can handle large and complex datasets with exceptional speed and accuracy. It also uses regularization techniques to prevent overfitting, allowing it to generalize well to new data. This is particularly important when working with high-dimensional datasets to reduce the risk of overfitting.

#### Random forest (RF)

Random Forest is an efficient supervised learning algorithm for classification and regression that works by combining multiple decision trees^68^. Each tree is trained on different subsets of data and random feature selection is performed, which increases the diversity of the model and reduces the risk of overfitting^69^. The algorithm is effective when working with large data sets and incomplete data, strengthening its generalization ability. Random Forests are preferred for solving complex problems by offering high accuracy and generalization capacity^70^.

#### CatBoost

CatBoost, as an innovative gradient boosting framework, distinguishes itself by its exceptional ability to process categorical features directly, which streamlines machine learning workflow and improves model accuracy^71^. Traditional machine learning algorithms often require extensive pre-processing steps such as one-hot encoding or label-encoding to convert categorical variables into numerical representations^72^. CatBoost eliminates this requirement by directly accepting categorical features as input using a sophisticated algorithm that learns the optimal splitting rules for these features during the training process^73^. This capability is particularly advantageous in datasets with a high proportion of categorical variables, as it reduces information loss and avoids the creation of high-dimensional feature spaces that can hinder model performance and scalability^74^.

#### Support vector machines (SVM)

Support Vector Machines (SVM) is a powerful supervised learning algorithm used for classification^75^ and regression tasks. This algorithm attempts to maximize the distance between data by creating a decision boundary or hyperplane that separates data points belonging to different classes^76^. The data points closest to the decision boundary are called support vectors and play an important role in defining the boundary^77^. When the data cannot be linearly separated, SVM uses a technique called the kernel trick to transform the data into a higher dimensional space, where a linear separation can be made^78^.

### Data mining method parameters

Table 3 presents the hyperparameters of the data mining methods used in this study. These parameters, which directly affect the performance and generalization capacity of each algorithm, were determined to optimize the interaction of the model with the data. In the MLP algorithm, the ‘relu’ activation function and the ‘adam’ optimization algorithm are preferred to learn non-linear relationships. In the Decision Tree algorithm, purity was measured with the ‘gini’ criterion and the maximum depth was not limited to increase the flexibility of the model. In the KNN method, the number of neighbours was set to 5 and the ‘minkowski’ metric was used for distance measurement. Although most parameters are left as default in the XGBoost algorithm, the ‘binary: logistic’ objective function and the ‘logloss’ evaluation metric are used to create a structure suitable for binary classification problems. In the Random Forest model, the number of trees was set to 100 and random variable subsets were selected in each tree with the ‘sqrt’ feature to increase the diversity and robustness of the model. The CatBoost algorithm was run with the default configuration and only the output detail level (‘verbose’) was specified. In the SVM algorithm, the kernel function ‘rbf’ was used to obtain nonlinear decision boundaries. The ‘C’ parameter was set to 1.0 to maintain the balance between regularization and classification. The parameters of all the methods used in the study were determined in accordance with the nature of the algorithm and the problem structure. They were structured in a way to maximize the model performance.

**Table 3:** Parameters for all applied data mining Methods.

Algorithm	Parameters
MLP	‘activation’: ‘relu’, ‘alpha’: 0.0001, ‘batch_size’: ‘auto’, ‘beta_1’: 0.9, ‘beta_2’: 0.999, ‘early_stopping’: False, ‘epsilon’: 1e-08, ‘hidden_layer_sizes’: (100,), ‘learning_rate’: ‘constant’, ‘learning_rate_init’: 0.001, ‘max_fun’: 15000, ‘max_iter’: 200, ‘momentum’: 0.9, ‘n_iter_no_change’: 10, ‘nesterovs_momentum’: True, ‘power_t’: 0.5, ‘random_state’: 42, ‘shuffle’: True, ‘solver’: ‘adam’, ‘tol’: 0.0001, ‘validation_fraction’: 0.1, ‘verbose’: False, ‘warm_start’: False
Decision Tree	‘ccp_alpha’: 0.0, ‘class_weight’: None, ‘criterion’: ‘gini’, ‘max_depth’: None, ‘max_features’: None, ‘max_leaf_nodes’: None, ‘min_impurity_decrease’: 0.0, ‘min_samples_leaf’: 1, ‘min_samples_split’: 2, ‘min_weight_fraction_leaf’: 0.0, ‘random_state’: 42, ‘splitter’: ‘best’
KNN	‘algorithm’: ‘auto’, ‘leaf_size’: 30, ‘metric’: ‘minkowski’, ‘metric_params’: None, ‘n_jobs’: None, ‘n_neighbors’: 5, ‘p’: 2, ‘weights’: ‘uniform’
XGBoost	‘objective’: ‘binary: logistic’, ‘base_score’: None, ‘booster’: None, ‘callbacks’: None, ‘colsample_bylevel’: None, ‘colsample_bynode’: None, ‘colsample_bytree’: None, ‘device’: None, ‘early_stopping_rounds’: None, ‘enable_categorical’: False, ‘eval_metric’: ‘logloss’, ‘feature_types’: None, ‘gamma’: None, ‘grow_policy’: None, ‘importance_type’: None, ‘interaction_constraints’: None, ‘learning_rate’: None, ‘max_bin’: None, ‘max_cat_threshold’: None, ‘max_cat_to_onehot’: None, ‘max_delta_step’: None, ‘max_depth’: None, ‘max_leaves’: None, ‘min_child_weight’: None, ‘missing’: nan, ‘monotone_constraints’: None, ‘multi_strategy’: None, ‘n_estimators’: None, ‘n_jobs’: None, ‘num_parallel_tree’: None, ‘random_state’: None, ‘reg_alpha’: None, ‘reg_lambda’: None, ‘sampling_method’: None, ‘scale_pos_weight’: None, ‘subsample’: None, ‘tree_method’: None, ‘validate_parameters’: None, ‘verbosity’: None, ‘use_label_encoder’: False
Random Forest	‘bootstrap’: True, ‘ccp_alpha’: 0.0, ‘class_weight’: None, ‘criterion’: ‘gini’, ‘max_depth’: None, ‘max_features’: ‘sqrt’, ‘max_leaf_nodes’: None, ‘max_samples’: None, ‘min_impurity_decrease’: 0.0, ‘min_samples_leaf’: 1, ‘min_samples_split’: 2, ‘min_weight_fraction_leaf’: 0.0, ‘n_estimators’: 100, ‘n_jobs’: None, ‘oob_score’: False, ‘random_state’: 42, ‘verbose’: 0, ‘warm_start’: False
CatBoost	‘verbose’: 0
SVM	‘C’: 1.0, ‘break_ties’: False, ‘cache_size’: 200, ‘class_weight’: None, ‘coef0’: 0.0, ‘decision_function_shape’: ‘ovr’, ‘degree’: 3, ‘gamma’: ‘scale’, ‘kernel’: ‘rbf’, ‘max_iter’: −1, ‘probability’: True, ‘random_state’: 42, ‘shrinking’: True, ‘tol’: 0.001, ‘verbose’: False

## Experimental results

In this study, university students’ attitudes towards artificial intelligence were evaluated over 1379 data collected through a scale application and classification process was carried out in three classes (Insufficient, Sufficient, Strongly Sufficient) using data mining methods such as MLP, Decision Tree, KNN, XGBoost, Random Forest, CatBoost and SVM algorithms. In the classification process, a 5-fold cross-validation method was applied, and the performance of each model was evaluated by basic performance metrics such as accuracy, precision, recall and F1 score calculated based on the confusion matrices obtained at the end of cross-validation.

The dataset used in the study exhibits a partial class imbalance. While accuracy is often considered sufficient for balanced datasets, in imbalanced datasets it is necessary to use additional metrics such as precision, recall, and F1-score to more accurately reflect model performance. For this reason, such metrics have been developed and are widely used in the literature. Taking this point into account, we did not limit ourselves to reporting only accuracy in the article; we also included precision, recall, and F1-score metrics for this reason.

### Results of MLP

**Fig. 3:**
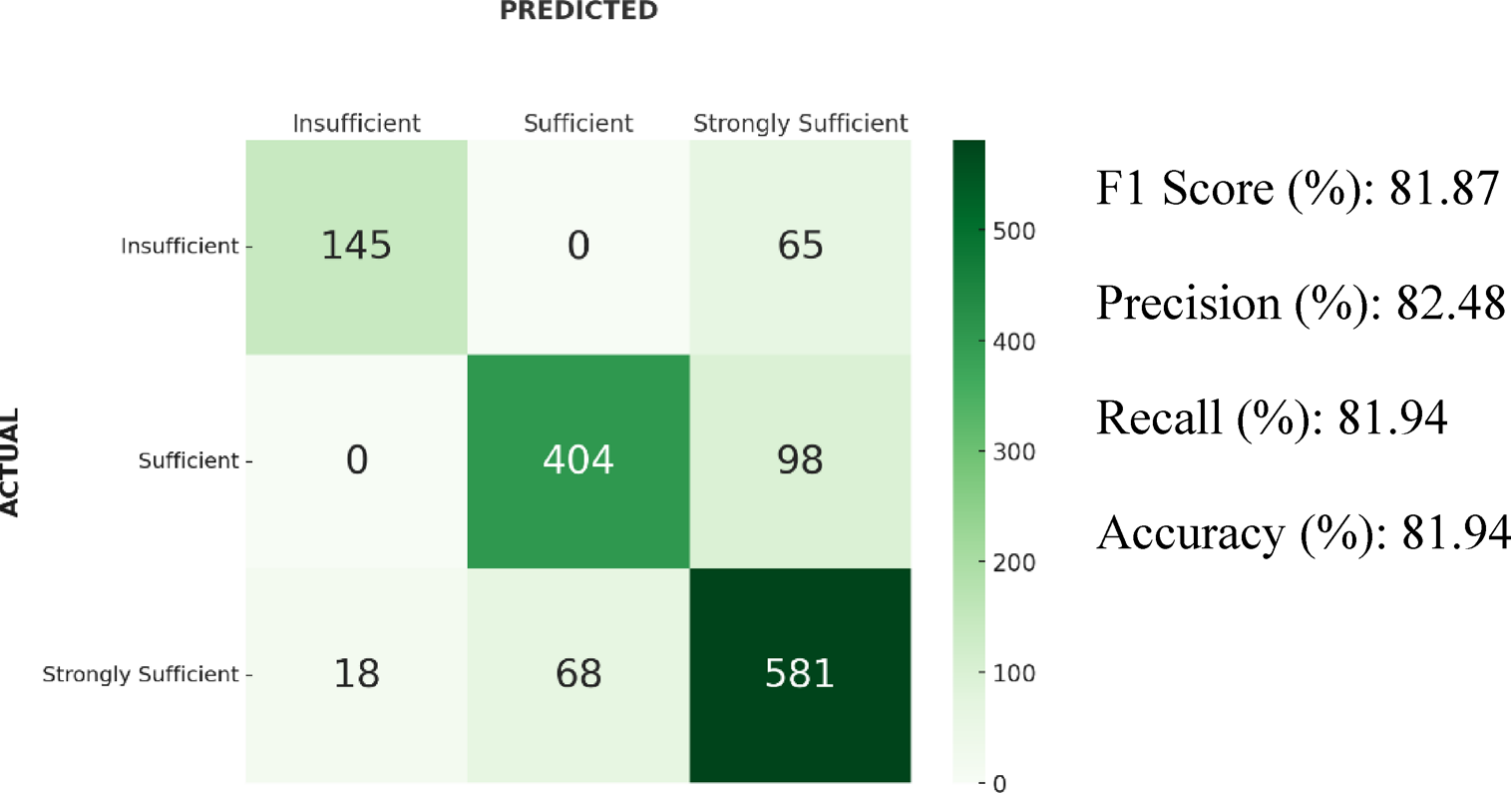
Confusion matrix and results of the MLP algorithm.

In the classification performed with the MLP algorithm, the 5-fold cross-validation resulted in an F1-score of 81.87%. In addition, the accuracy was 81.94%, precision 82.48%, and recall 81.94%. According to the confusion matrix, 65 instances from the “Insufficient” class and 98 instances from the “Sufficient” class were predicted as “Strongly Sufficient.” Furthermore, 68 samples from the “Strongly Sufficient” class were classified as “Sufficient,” and 18 were classified as “Insufficient”. Confusion matrix and results of the MLP algorithm is shown in Fig. 3.

### Results of decision tree

**Fig. 4:**
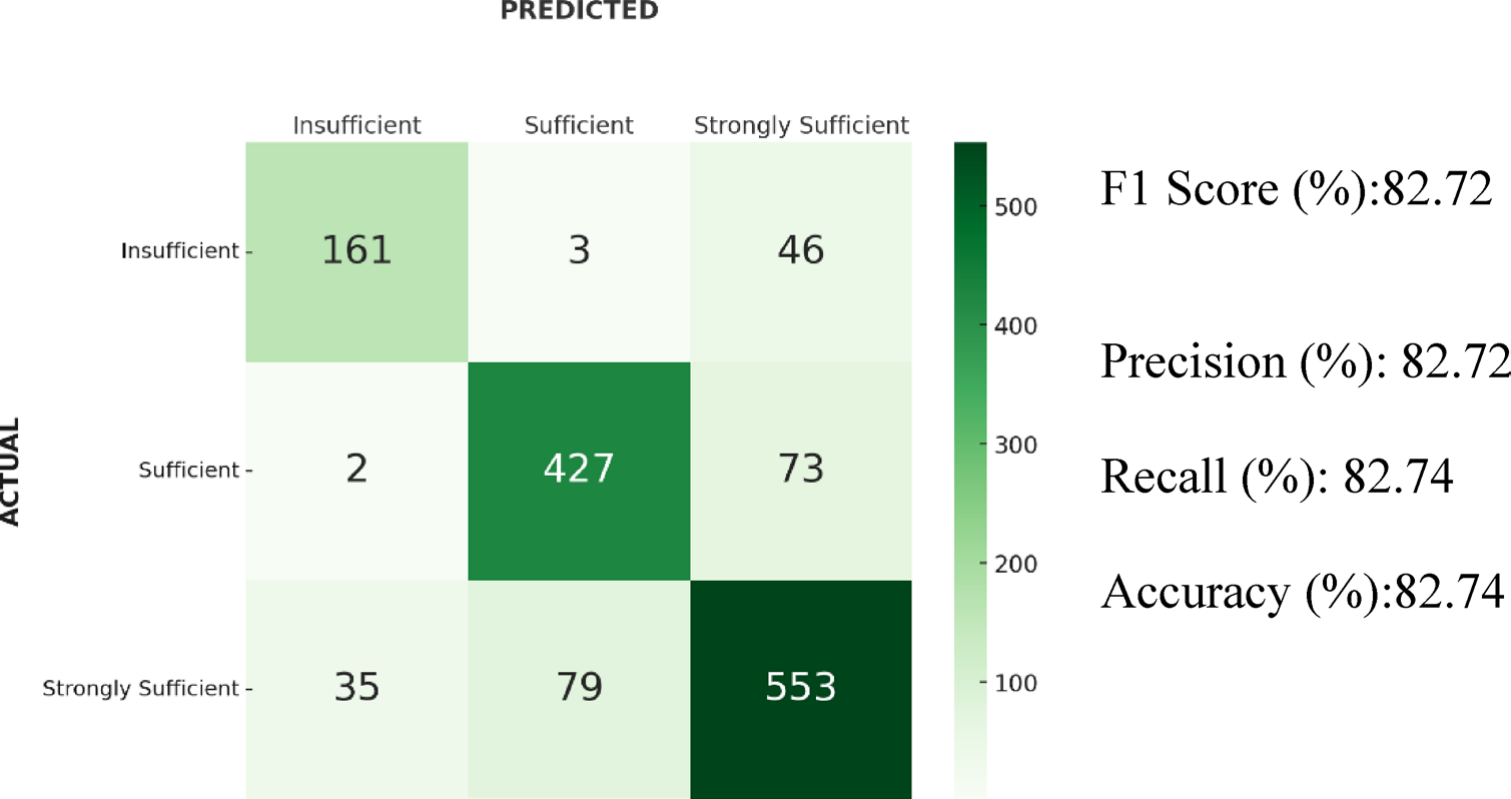
Confusion matrix and results of the Decision Tree algorithm.

In the Decision Tree algorithm, the F1-score was 82.72% based on 5-fold cross-validation. The accuracy was 82.74%, precision 82.72%, and recall 82.74%. The confusion matrix showed that 79 samples from the “Strongly Sufficient” class were classified as “Sufficient,” and 35 samples were classified as “Insufficient.” Additionally, 73 samples from the “Sufficient” class were predicted as “Strongly Sufficient,” while 46 samples from the “Insufficient” class were also predicted as “Strongly Sufficient”. Confusion matrix and results of the Decision Tree algorithm is shown in Fig. 4.

### Results of KNN

**Fig. 5:**
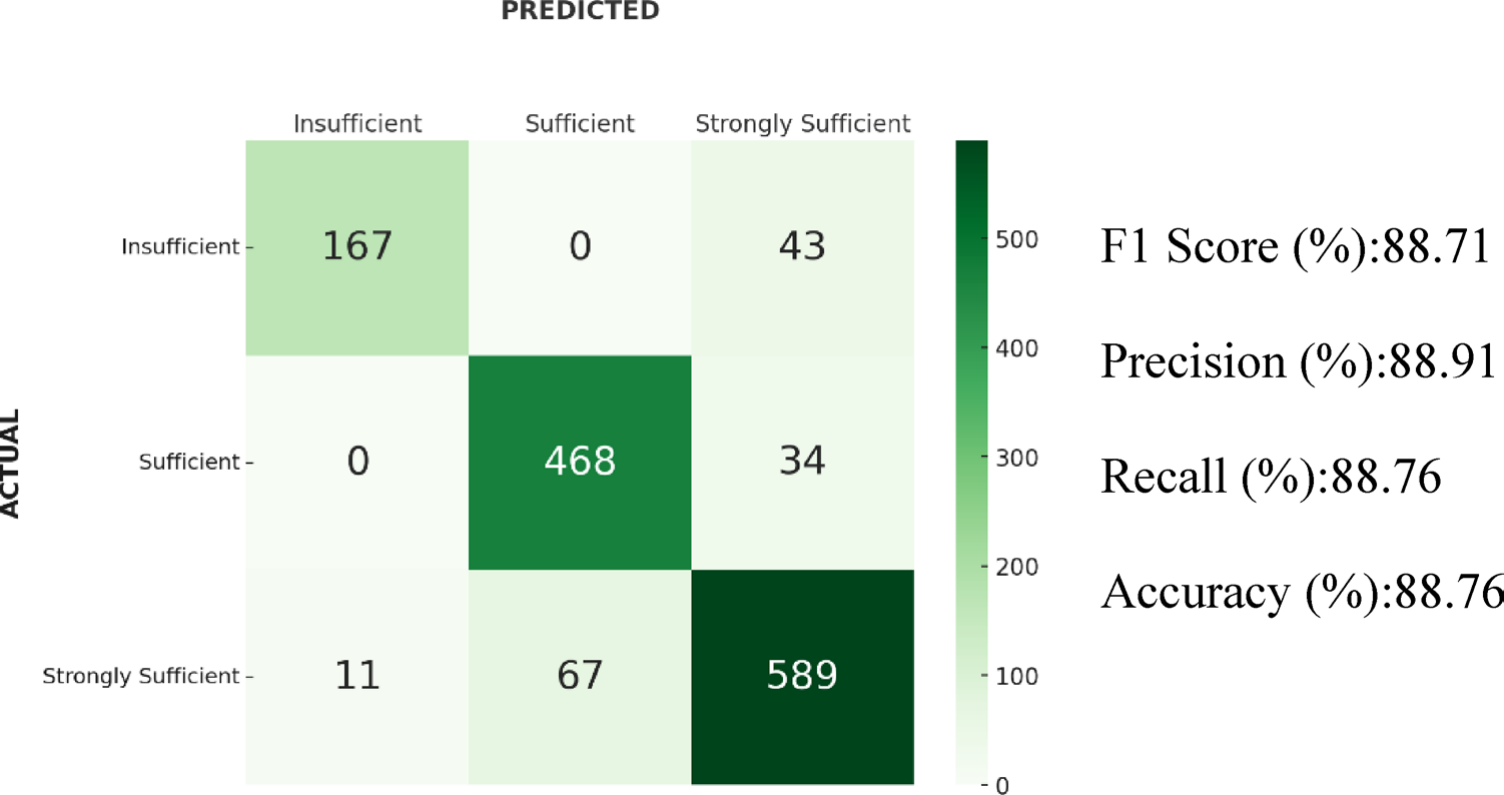
Confusion matrix and results of the KNN algorithm.

For the KNN algorithm, the F1-score was 88.71%. The accuracy was 88.76%, precision 88.91%, and recall 88.76%. The confusion matrix indicated that 67 samples from the “Strongly Sufficient” class were classified as “Sufficient” and 11 as “Insufficient.” Moreover, 34 samples from the “Sufficient” class were predicted as “Strongly Sufficient,” and 43 samples from the “Insufficient” class were also classified as “Strongly Sufficient”. Confusion matrix and results of the KNN algorithm is shown in Fig. 5.

### Results of XGBoost

**Fig. 6:**
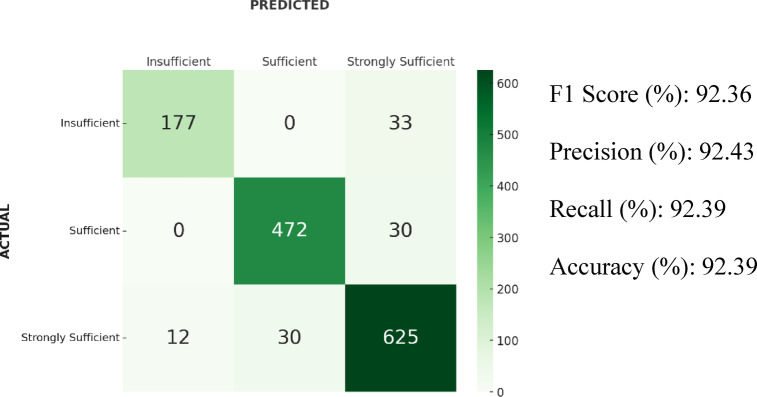
Confusion matrix and results of the XGBoost algorithm.

In the XGBoost algorithm, the F1-score was 92.36%. In addition, the accuracy was 92.39%, precision 92.43%, and recall 92.39%. According to the confusion matrix, 30 samples from the “Strongly Sufficient” class were classified as “Sufficient,” and 12 samples as “Insufficient.” Furthermore, 33 samples from the “Insufficient” class and 30 from the “Sufficient” class were predicted as “Strongly Sufficient”. Confusion matrix and results of the XGBoost algorithm is shown in Fig. 6.

### Results of random forest

**Fig. 7:**
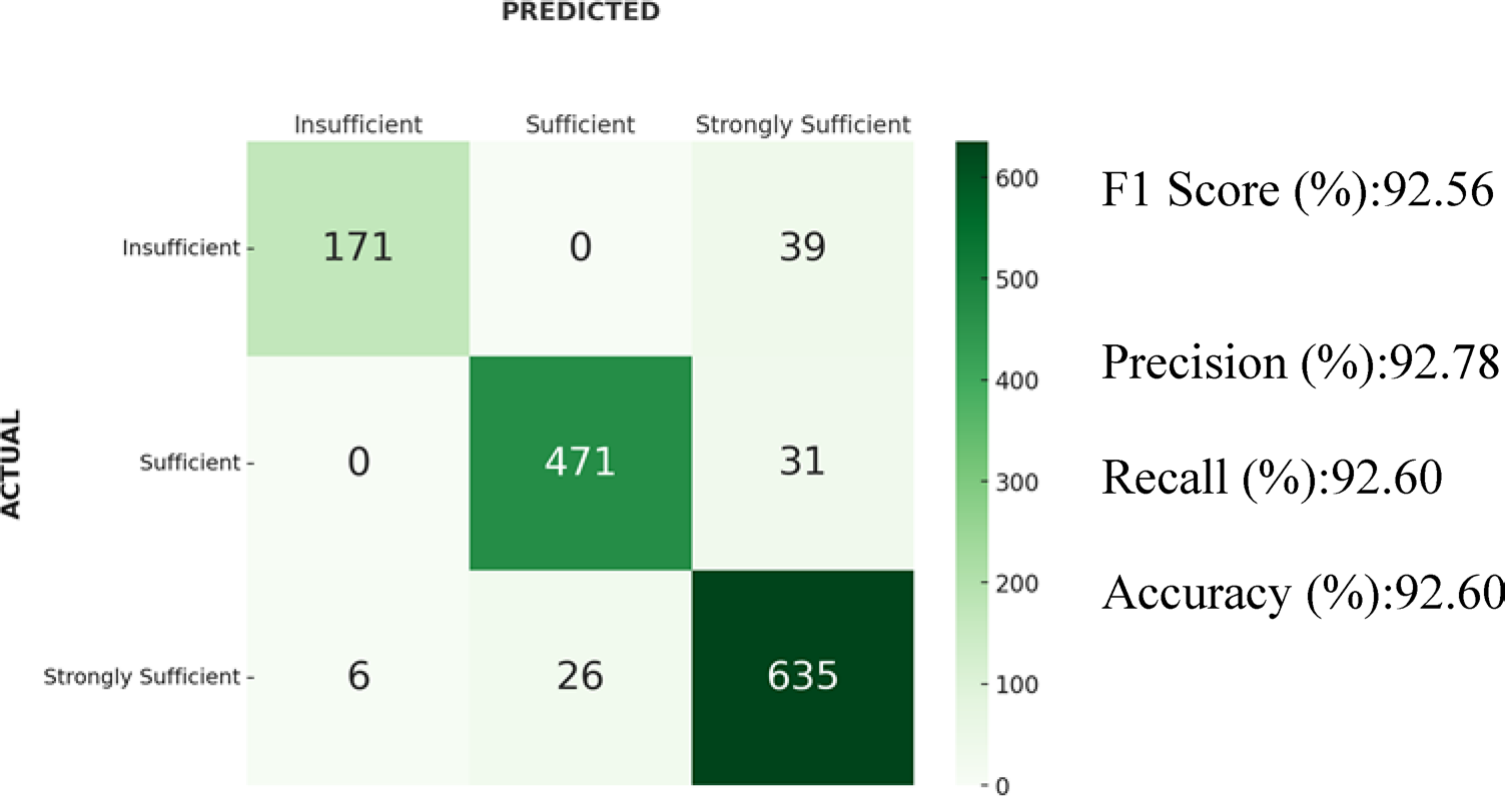
Confusion matrix and results of the Random Forest algorithm.

For the Random Forest algorithm, the F1-score was 92.56%. Additionally, the accuracy was 92.60%, precision 92.78%, and recall 92.60%. The confusion matrix showed that 26 samples from the “Strongly Sufficient” class were classified as “Sufficient” and 6 as “Insufficient.” In addition, 39 samples from the “Insufficient” class and 31 samples from the “Sufficient” class were classified as “Strongly Sufficient”. Confusion natrix and results of the Random Forest algorithm is shown in Fig. 7.

### Results of catboost

**Fig. 8:**
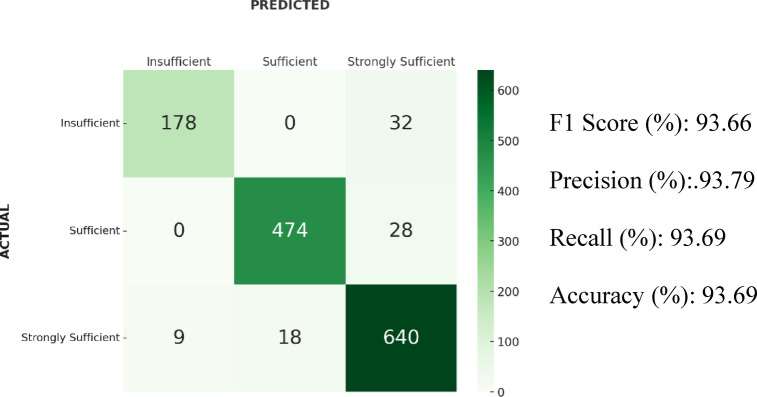
Confusion matrix and results of the CatBoost algorithm.

In the CatBoost algorithm, the F1-score was 93.66%. The accuracy was 93.69%, precision 93.79%, and recall 93.69%. According to the confusion matrix, 18 samples from the “Strongly Sufficient” class were classified as “Sufficient,” and 9 as “Insufficient.” Moreover, 32 samples from the “Insufficient” class and 28 samples from the “Sufficient” class were predicted as “Strongly Sufficient”. Confusion matrix and results of the CatBoost algorithm is shown in Fig. 8.

### Results of SVM

**Fig. 9:**
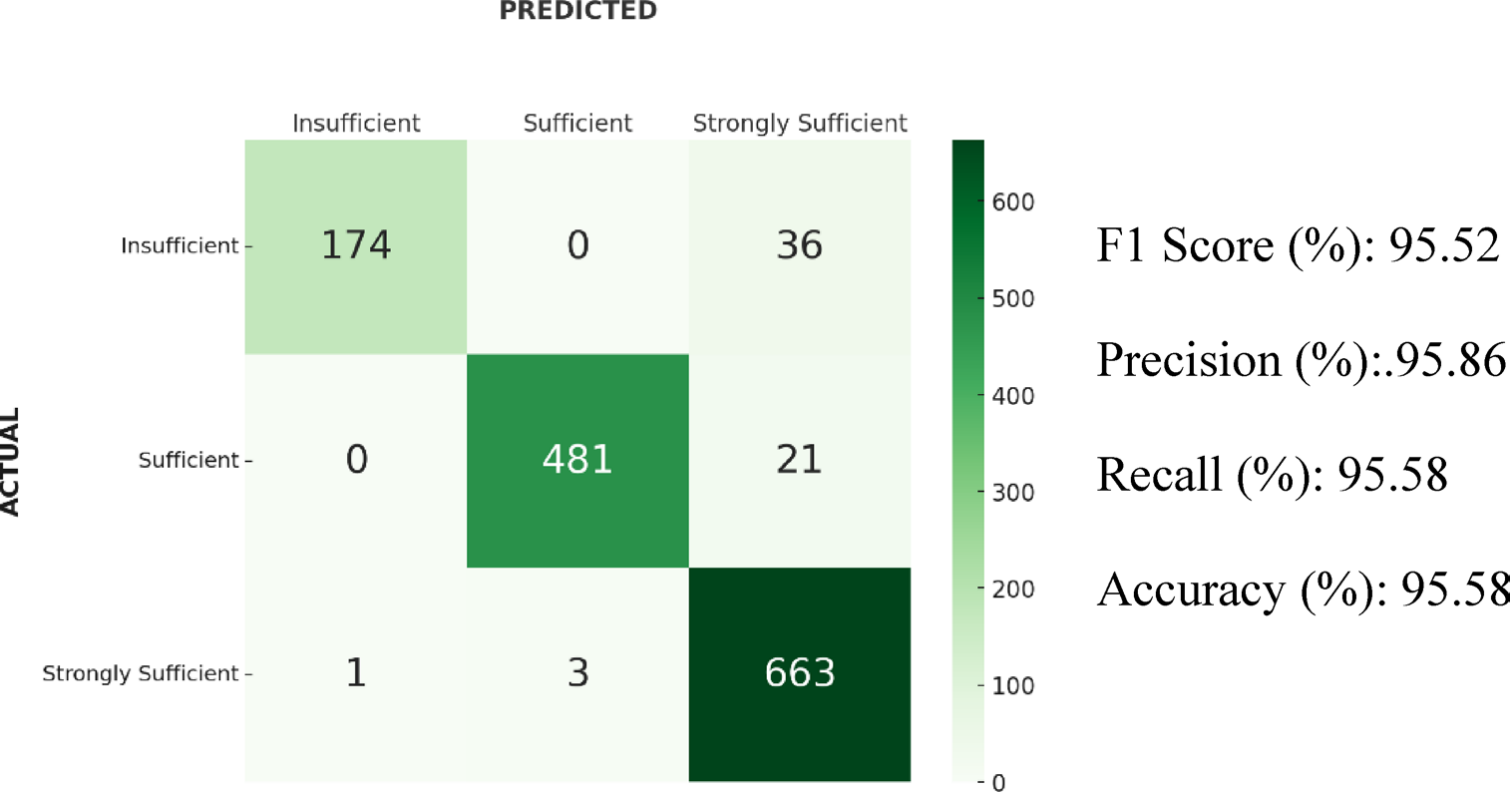
Confusion matrix and results of the SVM algorithm.

In the SVM algorithm, the highest performance was obtained with an F1-score of 95.52%. The accuracy was 95.58%, precision 95.86%, and recall 95.58%. The confusion matrix showed that 36 samples from the “Insufficient” class were classified as “Strongly Sufficient.” In addition, 21 samples from the “Sufficient” class were classified as “Strongly Sufficient,” while 3 samples from the “Strongly Sufficient” class were predicted as “Sufficient,” and 1 sample was classified as “Insufficient”. Confusion matrix and results of the SVM algorithm is shown in Fig. 9.

## Conclusions and discussion

In this study, university students’ attitudes towards artificial intelligence were evaluated over 1379 data collected through a scale application. Data was classified into three classes (Insufficient, Sufficient, Strongly Sufficient) using data mining methods. Performance metrics such as accuracy, precision, recall, and F1 score were calculated for each method. Table 4 shows the calculations.

**Table 4:** Performance metric results of data mining Methods.

Method	F1-Score (%)	Precision (%)	Recall (%)	Accuracy (%)
MLP	81.87	82.48	81.94	81.94
Decision Tree	82.72	82.72	82.74	82.74
KNN	88.71	88.91	88.76	88.76
XGBoost	92.36	92.43	92.39	92.39
Random Forest	92.56	92.78	92.60	92.60
CatBoost	93.66	93.79	93.69	93.69
SVM	95.52	95.86	95.58	95.58

When comparing the models based on the F1-score, which is a more reliable metric in the presence of class imbalance, the results show clear differences in performance:

- The lowest F1-score was achieved by the MLP algorithm (81.87%), indicating weaker performance compared to the other models.
- The Decision Tree algorithm produced an F1-score of 82.72%, only slightly higher than MLP, showing modest improvement.
- The KNN algorithm achieved an F1-score of 88.71%, reflecting better balance across the classes.
- More advanced ensemble methods provided stronger results: XGBoost (92.36%), Random Forest (92.56%), and CatBoost (93.66%) all achieved F1-scores above 92%, showing high classification performance.
- The SVM algorithm obtained the highest F1-score of 95.52%, demonstrating the best overall performance among all models.

Overall, the results highlight that while accuracy values are high across most models, the F1-score provides a more meaningful measure of performance in this study, particularly considering the partial class imbalance present in the dataset.

When the confusion matrixes were analysed, it was observed that confusion occurred especially in the Strongly Sufficient class. The fact that this class is frequently classified as Sufficient or Insufficient indicates that the transitivity between students’ attitude levels is high. This is a challenging factor for classification models.

In general, it is concluded that the data mining methods applied in the study provide effective tools for analyzing students’ attitudes towards artificial intelligence. Advanced methods such as SVM, CatBoost, Random Forest and XGBoost provide reliable results in attitude analysis with high success rates. The fact that these methods make visible not only classification success, but also transition and error analysis between classes, offers in-depth evaluation opportunities for researchers.

Pairwise Wilcoxon signed-rank tests were conducted to assess the statistical significance of performance differences among the seven algorithms. As shown in Table 5, most model pairs exhibit statistically significant differences (*p* < 0.05). Notably, the comparison between XGBoost and RF did not show a significant difference (*p* = 1.0), indicating similar performance. These results confirm that, except for specific cases, the algorithms perform significantly differently on the dataset.

**Table 5:** Pairwise Wilcoxon signed-rank test results for algorithm comparisons.

Model 1	Model 2	*p*-value
MLP	DT	0,02
MLP	KNN	0,01
MLP	XGBoost	0,01
MLP	RF	0,01
MLP	CatBoost	0,01
MLP	SVM	0,01
DT	KNN	0,01
DT	XGBoost	0,01
DT	RF	0,01
DT	CatBoost	0,01
DT	SVM	0,01
KNN	XGBoost	0,01
KNN	RF	0,01
KNN	CatBoost	0,01
KNN	SVM	0,01
XGBoost	RF	1
XGBoost	CatBoost	0,03
XGBoost	SVM	0,01
RF	CatBoost	0,01
RF	SVM	0,01
CatBoost	SVM	0,01

*p* < 0,05.

Following the pairwise Wilcoxon signed-rank tests for the seven algorithms (MLP, Decision Tree, kNN, XGBoost, Random Forest, CatBoost, and SVM), the relative importance of the input features was analyzed to provide actionable insights into the factors influencing students’ attitudes toward AI. Table 6 presents the ranked feature importance, both overall and algorithm-specific. The first column shows the overall ranking of features from the most to the least important. Columns 2 through 5 display the importance scores as determined by the general evaluation (overall), Random Forest, SVM, and Boosting algorithms, respectively.

**Table 6:** Feature importance Rankings.

Importance Rank	Global Ranking (InfoGain)	RF	SVM	Boosting Models (XGBoost/CatBoost)
1	23	13	16	23
2	18	17	17	17
3	19	23	15	10
4	17	15	22	18
5	8	8	21	6
6	13	19	11	25
7	21	3	23	4
8	20	20	18	21
9	2	10	8	19
10	25	4	25	26
11	5	21	7	22
12	22	6	9	2
13	26	25	20	15
14	6	11	12	5
15	11	18	19	13
16	7	5	24	7
17	15	22	26	11
18	10	2	4	8
19	3	26	5	20
20	12	7	10	9
21	9	12	6	12
22	1	16	1	24
23	4	1	3	14
24	14	9	2	1
25	16	14	14	3
26	24	24	13	16

As illustrated in Table 6, certain features consistently appear among the top-ranked across multiple algorithms, indicating their strong influence on the prediction of students’ attitudes. These results not only complement the performance comparisons reported earlier but also offer valuable information for designing targeted interventions and educational programs aimed at improving AI literacy.

The findings of the study reveal that data mining methods offer a powerful methodological framework for analysing educational data. The applicability and reliability of these methods in understanding university students’ attitudes towards artificial intelligence were found to be high. These analyses, which can serve purposes such as shaping educational policies, improving teaching processes and increasing technological awareness, can serve as an example for studies in different fields.

In future studies, including demographic variables (gender, field of study, grade level, etc.) in the data set may increase analysis depth. In addition, the use of data balancing techniques to reduce the imbalance between classes is an important step that can increase the model’s success. Similar studies conducted in different institutions and sample groups will contribute to testing the generalizability of the results obtained.

This study has shown that data mining methods can be used successfully and effectively in evaluating university students’ attitudes towards artificial intelligence. The findings are both methodologically and conceptually guiding future academic research and applied analysis in this field.

Table 6 illustrates the ranked feature importance across all evaluated algorithms. Certain features consistently appear among the top-ranked across multiple models, indicating their strong influence on predicting students’ attitudes toward AI. Notably, the features “I am interested in the development of AI”, “I want to work in the field of AI”, and “I like using objects related to AI” were frequently ranked highest in Random Forest, Boosting, and SVM models. This suggests that students’ personal interest in AI, career intentions, and engagement with AI-related tools are critical determinants of their attitudes toward AI.

Moreover, the alignment between these top-ranked features and the predictive performance of the respective models emphasizes the reliability of these findings. The consistency of these features across algorithms provides actionable insights for educational interventions, such as designing targeted modules to foster AI interest, support AI-related career guidance, or enhance hands-on experience with AI tools. These insights complement the earlier model performance comparisons and strengthen the practical implications of the study.

While deep learning models have shown remarkable results in various domains, their application in this study was limited by the dataset characteristics. The structured nature and moderate size of the dataset (33 numerical features, *n* = 1379) favored traditional machine learning algorithms, which provide more interpretable results and are less prone to overfitting in such contexts. Future research could investigate the potential benefits of deep learning approaches when larger or multimodal datasets, such as text or image data, become available.

The findings of this study not only contribute to the classification of attitudes towards artificial intelligence but also indicate a potential for integration with high-tech applications in education. In particular, digital learning environments equipped with technologies such as big data and the Internet of Things (IoT) continuously collect large volumes of data from students. In such environments, the data mining techniques used in this study can be effectively employed to analyze students’ attitudes toward artificial intelligence in real-time, detect changes in trends, and adapt instructional strategies accordingly.

Within this framework, artificial intelligence systems enable a more holistic understanding of the learning process by analyzing not only cognitive but also emotional components. Through emotional patterns extracted from learning data, students’ motivation levels, attention spans, and cognitive load can be identified, which contributes to tailoring instructional processes to individual needs. Especially with the integration of techniques such as machine learning, facial recognition, and emotion analysis into educational settings, it becomes possible to deliver content suited to students’ momentary emotional states and provide real-time feedback. Such AI-based systems offer significant contributions to the creation of flexible and personalized learning environments that consider students’ emotional and cognitive needs.

Therefore, the data mining models used in this study are valuable not only for attitude classification but also for establishing decision support mechanisms and designing personalized learning processes in technologically advanced educational environments. Future studies with larger-scale and multimodal datasets may further enhance the impact of such AI-supported analyses.

## Data Availability

The datasets used and/or analysed during the current study available from the corresponding author on reasonable request. The data used to support the findings of this study are available on the https://citedata.com/ATAID_Dataset.zip.
